# Modeling the Influence of Chronic Sleep Restriction on Cortisol Circadian Rhythms, with Implications for Metabolic Disorders

**DOI:** 10.3390/metabo11080483

**Published:** 2021-07-27

**Authors:** Rohit Rao, Pramod Somvanshi, Elizabeth B. Klerman, Charles Marmar, Francis J. Doyle

**Affiliations:** 1 Harvard John A. Paulson School of Engineering and Applied Sciences, Harvard University, Cambridge, MA 02139, USA; rohitrao@g.harvard.edu (R.R.); pramodrs@uohyd.ac.in (P.S.); 2 Department of Systems and Computational Biology, School of Life Sciences, University of Hyderabad, Hyderabad 500046, India; 3 Division of Sleep Medicine, Harvard Medical School, Boston, MA 02115, USA; elizabeth_klerman@hms.harvard.edu; 4 Department of Neurology, Massachusetts General Hospital, Boston, MA 02114, USA; 5 Department of Psychiatry, New York Langone Medical School, New York, NY 10016, USA; charles.marmar@nyulangone.edu

**Keywords:** mathematical modeling, HPA axis, cortisol, sleep/wake cycle, sleep restriction, circadian rhythms

## Abstract

Chronic sleep deficiency is prevalent in modern society and is associated with increased risk of metabolic and other diseases. While the mechanisms by which chronic sleep deficiency induces pathophysiological changes are yet to be elucidated, the hypothalamic–pituitary–adrenal (HPA) axis may be an important mediator of these effects. Cortisol, the primary hormone of the HPA axis, exhibits robust circadian rhythmicity and is moderately influenced by sleep and wake states and other physiology. Several studies have explored the effects of acute or chronic sleep deficiency (i.e., usually from self-selected chronic sleep restriction, CSR) on the HPA axis. Quantifying long-term changes in the circadian rhythm of cortisol under CSR in controlled conditions is inadequately studied due to practical limitations. We use a semi-mechanistic mathematical model of the HPA axis and the sleep/wake cycle to explore the influence of CSR on cortisol circadian rhythmicity. In qualitative agreement with experimental findings, model simulations predict that CSR results in physiologically relevant disruptions in the phase and amplitude of the cortisol rhythm. The mathematical model presented in this work provides a mechanistic framework to further explore how CSR might lead to HPA axis disruption and subsequent development of chronic metabolic complications.

## 1. Introduction

Adequate sleep is essential to the maintenance of metabolic homeostasis [1,2]. Epidemiological studies have implicated sleep disruption and changes in sleep duration and sleep disturbances in the development of numerous health disorders. Shorter sleep duration and disrupted sleep have been associated with increased all-cause mortality [3,4]; increased risk of cardiovascular death [4], Type II diabetes, metabolic syndrome [5], obesity, and hypertension [6]; accelerated aging-associated cognitive function decline [7]; vulnerability to infection [8].

The physiological mechanisms by which reduced sleep (i.e., sleep deficiency) contributes to pathophysiological metabolic impairments are not entirely understood. The hypothalamic–pituitary–adrenal (HPA) axis, an important signaling system primarily regulating the stress response, is one putative mediator of the physiological effects associated with homeostatic (from habitual sleep timing and duration) and disrupted (from restricted or irregular sleep timing and duration) sleep and wake patterns [9,10,11]. The HPA axis is composed of signaling interactions among three primary hormones—CRH, ACTH, and the glucocorticoids, primarily cortisol in humans—that form an autoregulatory negative feedback loop. CRH from the hypothalamus induces the secretion of ACTH from the pituitary, which further induces the de novo biosynthesis and secretion of cortisol from the adrenal glands. Cortisol subsequently, negatively regulates the secretion of CRH and ACTH primarily through receptor-mediated mechanisms [12]. Cortisol binds to its receptors to regulate multiple metabolic functions including glucose metabolism, free fatty acid release from white adipose tissue, and protein catabolism [13]. Additionally, receptor-mediated cortisol signaling also regulates other critical physiological effects such as the stress and immune responses, and cardiovascular function [13]. The hormones of the HPA axis exhibit regular approximately 24 h periodic rhythms that are primarily driven by the central circadian clock located in the hypothalamic suprachiasmatic nuclei (SCN). For sleep occurring at “normal” times, circulating glucocorticoid levels exhibit a peak around the onset of awakening, and thereafter decline to approach a minimum during sleep [10].

Sleep has a comparatively moderate but reproducible inhibitory effect on HPA axis activity which can be observed under some circumstances [14]. Under free-running conditions (i.e., in the absence of entraining environmental cues), sleep onset occurs at a later circadian time that is after the start of rising phase of cortisol, and sleep onset inhibits the rise in cortisol levels [15,16]. Sleep onset during habitual wake times (i.e., daytime sleep in humans) has a transient inhibitory influence on cortisol secretion [17]. Transient nocturnal awakenings are associated with secretory cortisol pulses [18]. Variations in slow wave activity (a marker for slow wave sleep) are negatively correlated with pulsatile cortisol secretion [19]. Chronic sleep restriction (CSR) disrupts HPA axis circadian rhythmicity, with elevated evening cortisol levels being observed most commonly [20,21,22].

The prevalence of CSR has become widespread in modern society. Epidemiological studies report an approximately 2 h decrease in sleep duration over the past 40 years: the average sleep duration in U.S. populations has fallen below ~7 h per night [23,24]. Epidemiological evidence shows an association between CSR and an increased risk for the development of obesity, type II diabetes, and similar cardiometabolic diseases [23,25]. Chronically restricted sleep durations and disrupted sleep often associated with modern lifestyles may have adverse health consequences partially from exposure to light at night and the associated circadian disturbances in important physiological mediators such as cortisol [26]. Systematic reviews of laboratory-based and randomized controlled studies have shown that sleep restriction-induced hormonal (e.g., the HPA axis) dysregulation is a potential causal mediator of associated metabolic disruption [27,28,29,30]. For example, CSR-associated elevations in evening cortisol levels and dampening of 24 h cortisol rhythm amplitudes are hypothesized to have maladaptive effects on carbohydrate regulation and adiposity, ultimately leading to the development of chronic metabolic disorders [30]. While cortisol, and other physiology, may be differentially affected by sleep deprivation (one extended wake episode), CSR (multiple nights of insufficient sleep), and disrupted sleep (multiple awakenings during the sleep episode), we chose to focus here on CSR.

Mathematical modeling of the relationship between sleep and cortisol activity can provide a framework to generate and test hypotheses regarding interventions that are not feasible to perform in controlled laboratory settings or in the real world. While prior studies have used a mathematical modeling framework to explore the effects of acute sleep deprivation on glucocorticoid rhythms [31,32], the effects of CSR on HPA axis activity are yet to be explored. In this work, we present a prototype mathematical model that accounts for the dual influence of both circadian and sleep/wake processes on HPA axis activity, thereby enabling a more physiologically relevant description of the effects of CSR on cortisol. The semi-mechanistic nature of the interactions captured by the model further enables a consideration of the influence of environmental disruption associated with modern lifestyles, (e.g., evening artificial light exposure) on the circadian dynamics of cortisol.

## 2. Results

**Figure 1:**
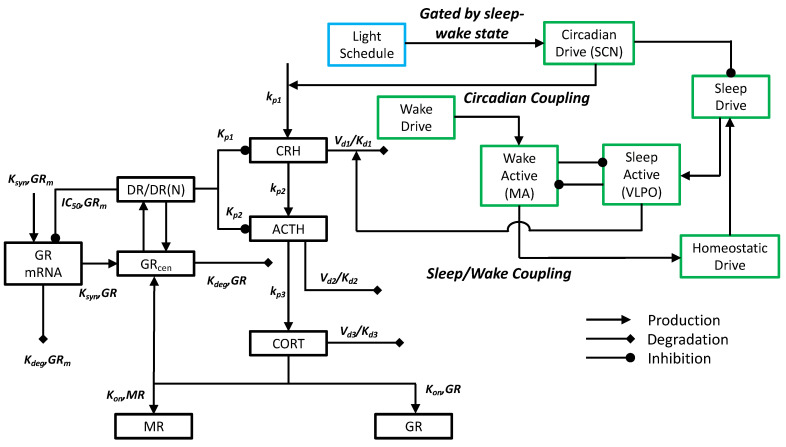
Schematic of our mathematical model: a physiologically based mathematical model of sleep/wake regulation coupled to a semi-mechanistic mathematical model of the circadian dynamics of the HPA axis. The physiologically based model of sleep/wake regulation accounts for the interactions between sleep-active VLPO (ventrolateral preoptic) neuronal population and the wake-active MA (monoaminergic) neuronal population. The homeostatic sleep drive and the wake drive both regulate the firing rates of the VLPO and MA neuronal populations, respectively. The entraining influence of light on the circadian rhythm is gated such that it influences the circadian drive from the SCN (suprachiasmatic nucleus) only if light exposure is present during waking hours. The circadian drive entrains the HPA axis (CRH, ACTH, cortisol (CORT), and GR-mediated negative feedback), which subsequently regulates the engagement of downstream, tissue glucocorticoid (GR) and mineralocorticoid (MR) receptors.

The mathematical model used in this work to explore the influence of sleep and wake timing and duration on the circadian activity of the HPA axis encompasses four physiologic elements: (i) a model composed of a phenomenological sleep/wake switch that captures the mutual inhibition of sleep-promoting ventrolateral preoptic area (VLPO) and wake-promoting monoaminergic neuronal populations leading to the emergence of sleep and wake states; (ii) the endogenous circadian drive (representative of the influence of the circadian pacemaker in the hypothalamic suprachiasmatic nucleus) and a homeostatic sleep drive (representative of “pressure” to sleep after being awake), which are integrated in the VLPO and thereby influence the sleep/wake switch; (iii) a self-regulatory negative feedback loop incorporating the interactions among the major hormones of the HPA axis, namely CRH, ACTH, and cortisol; (iv) an external light signal representative of the entraining influence of environment light on the endogenous circadian clock (and subsequently circadian rhythms of the glucocorticoids and the sleep/wake switch). A schematic of the model components and their interactions is provided in Figure 1. The homeostatic sleep drive mentioned in Element (ii) is phenomenologically representative of sleep pressure or sleep debt, which increases with time spent awake [33]. Details regarding the mathematical equations describing the physiological model of sleep/wake regulation and that of the HPA axis are provided in the Materials and Methods sections.

**Figure 2:**
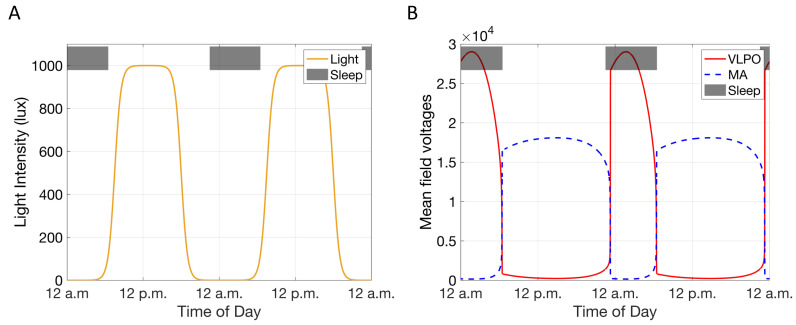
(**A**) Forty-eight hours of the nominal light schedule [maximal light intensity of 1000 lux after simulated sunrise (6 a.m.) and a minimal light intensity of 0 lux after simulated sunset (6 p.m.)] used for model simulations. (**B**) The mean-field firing rates of the wake-active, MA (blue), and sleep-active, VLPO (red) populations across 48 h. The model describes a simulated sleep-state (black shaded bars at top of panels) when the mean firing rate for the sleep-active population (VLPO) is greater than that of the wake-active neuronal population (MA) and describes a simulated wake-state (no shaded bars at top of panel) when the firing rate for the wake-active population is greater than that of the sleep-active population.

Under the influence of an idealized light schedule i.e., lights ON from approximately 6 a.m. (dawn) to approximately 6 p.m. (dusk) with a maximal light intensity of 1000 lux between 6 a.m. and 6 p.m. and a minimal light intensity of 0 lux otherwise, the dynamics of the model result in a habitual sleep schedule with a wake time of just before 7 a.m., and sleep time approximately 11 p.m. (Condition a) (Figure 2). We refer to this idealized light schedule and the resultant model dynamics as the “nominal” case in the following text and is similar to light schedules used previously to model sleep schedules prevalent in hunter gatherer societies [34] and in rodent studies. The model also has the flexibility to represent light schedules more typically observed by humans in modern day societies, where there is substantial exposure to evening light.

Entrainment of the physiological model of the sleep/wake cycle by the nominal light schedule results in a mean-field firing rate of the simulated wake-active and sleep-active neuronal populations, which determine the sleep and wake states of the model, respectively. More specifically, the model describes a wake state when the mean-field firing rate of the wake-promoting monoaminergic neuronal population is greater than that of the sleep-promoting VLPO population, and a sleep state when the converse is true (Figure 2).

**Figure 3:**
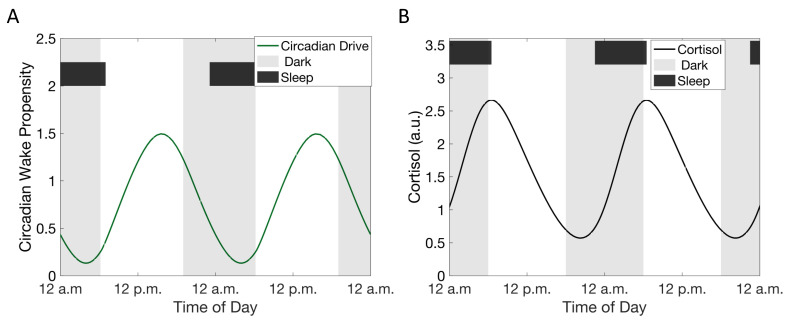
(**A**) The circadian drive (green) of the physiological sleep-wake model that regulates the homeostatic drive and the secretion of CRH in a time-of-day dependent manner. (**B**) The cortisol circadian rhythm (black) given the shown sleep (black shaded bars at top of panels) and light-dark (white and grey shaded regions, respectively) schedules.

Under the influence of the nominal light schedule, the model parameters are calibrated such that the circadian drive peaks in the middle of the day [35] (Figure 3) and the model of the HPA axis produces a circadian rhythm of cortisol (Figure 3), ACTH, and CRH (Figure S1). The phase adopted by the cortisol rhythm under the influence of the nominal light schedule and the model generated sleep-schedule closely follows physiological observations [9]. The cortisol rhythm peaks shortly after waking, soon after the beginning of light exposure [9]. The rising phase of the rhythm is shorter than the falling phase, with nadir of the rhythm occurring shortly before the onset of sleep [10,36], in qualitative agreement with experimental observations.

**Figure 4:**
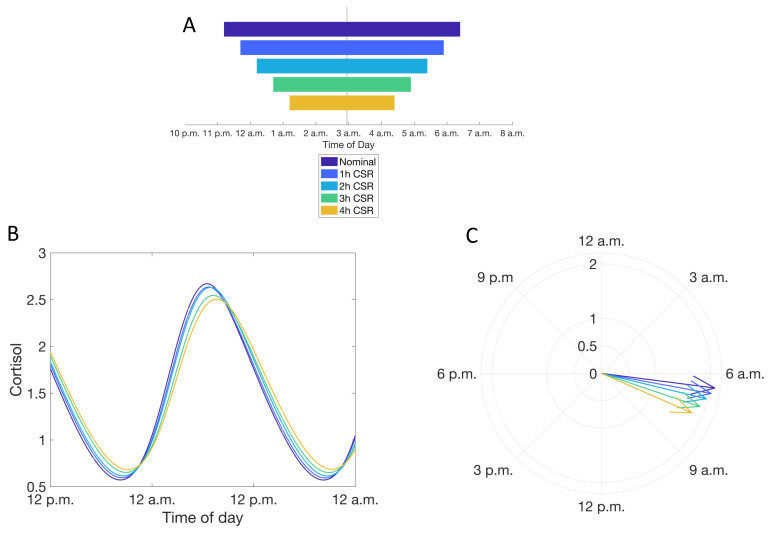
Simulating the influence of chronic sleep restriction (CSR) on the circadian rhythms of cortisol. (**A**) Simulated CSR schedules used. (**B**) Cortisol circadian rhythms with increasing levels of CSR. Color of line reflects schedule in (A). (**C**) Phase of the cortisol rhythm with different levels of CSR. Color of line reflects schedule in (A).

We specifically investigated the features of the cortisol rhythm after adaptation to CSR, rather than analyzing transient changes in the glucocorticoid rhythm in response to irregular or continually changing sleep schedules. CSR was imposed through both a delay in sleep time and an equal duration advance in wake time (Figure 4, Condition b, see Materials and Methods); this results in a delay in the phase of the cortisol rhythms. The phase delay in the cortisol rhythm is accompanied by an increase in the minima and a decrease in the peak levels of the cortisol rhythm leading to a dampening of the amplitude of the rhythm. These changes in the cortisol rhythm become more pronounced with increasing levels of CSR: increasing the duration of CSR increases the minima of cortisol levels and results in a greater delay in the timing of the peak of the cortisol circadian rhythm (Figure 4).

**Figure 5:**
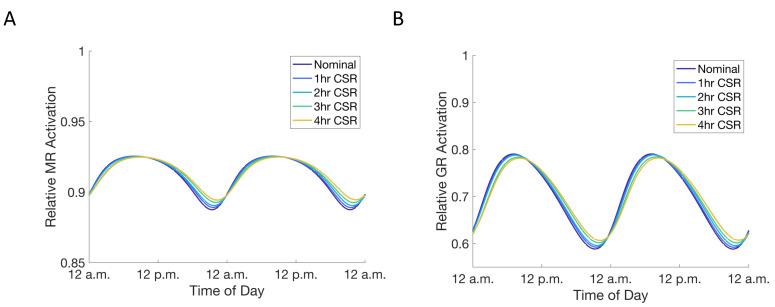
(**A**,**B**) Increase in cortisol minima is associated with a change in relative activation of GR (**B**) and MR (**A**) receptors. Colors as in Figure 4A.

We subsequently modeled the dynamics of the receptor–glucocorticoid complex during habitual and CSR conditions, specifically exploring how sleep influences the two primary receptors that bind the glucocorticoids, namely the high-affinity mineralocorticoid receptor (MR) and the lower-affinity glucocorticoid receptor (GR). Studying the dynamics of cortisol-bound GR activity could provide insights into how changes in glucocorticoid levels influence signal transduction to downstream glucocorticoid response elements (GRE). The fraction of the total receptor pool bound to cortisol and able to transduce receptor-mediated effects to GRE depends on condition (Figure 5). In the case of the cortisol profile under the light schedule, both the GR and MR reach maximal occupancy during the circadian peak of the cortisol rhythm. However, given the higher affinity of the MR, the majority of these receptors are occupied even at the circadian nadir of the cortisol rhythm. In contrast, only about half the GR are occupied at the cortisol circadian nadir. These dynamics are in qualitative agreement with experimental observations, where MR exhibit high occupancy even at physiologically low levels of cortisol, given their much higher affinity for glucocorticoids [37,38]. The dampening of the circadian amplitude of the cortisol rhythm in response to increasing levels of CSR results in a dampening of the receptor amplitude. The increase in nadir levels of cortisol due to CSR results in almost complete occupancy of the MR even at the minima of the cortisol rhythm.

**Figure 6:**
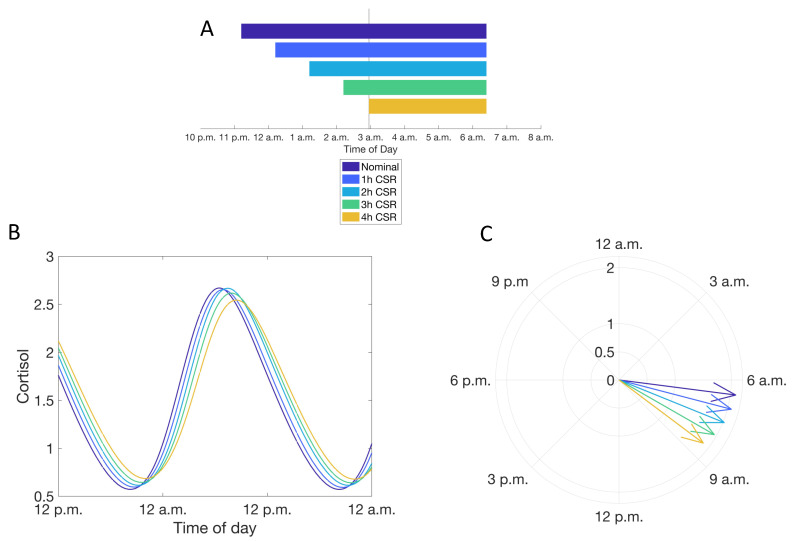
(**A**–**C**) Conditions as in Figure 5, respectively, except the influence of CSR is adjusted by changing the scheduled sleep onset time.

**Figure 7:**
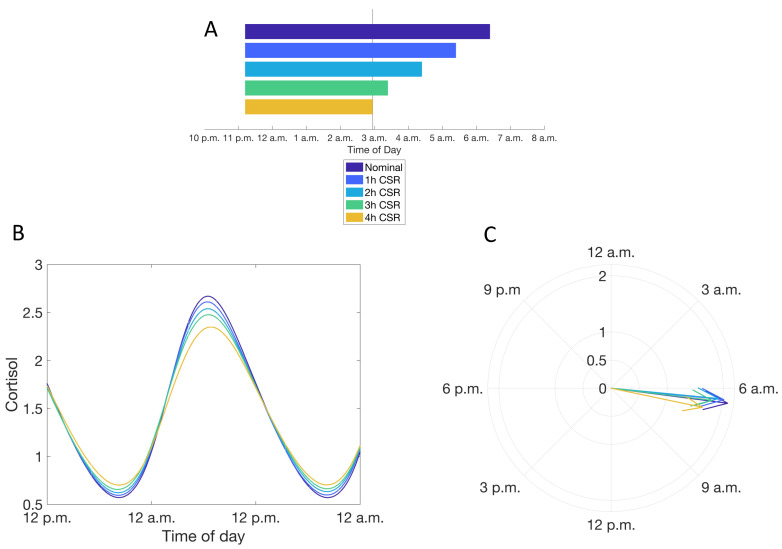
(**A**–**C**) Conditions as in Figure 5, respectively, except the CSR is adjusted by changing the scheduled wake time.

We then explored how CSR as a result of exclusively changing the sleep time (Condition b2) differentially influenced the circadian dynamics of cortisol in comparison to the CSR as a result of a change in the wake time only (Condition b3). Incrementally delaying the sleep time while maintaining the habitual wake time (as occurs when there are scheduled morning activities such as work or school) resulted in a substantially phase delay as well as a dampening of the cortisol circadian rhythm. The change in phase of the rhythm was more pronounced in comparison to when CSR was imposed by simultaneously changing both sleep and wake times (Condition b1) (Figure 6). Interestingly, in contrast, CSR due to an advance in the wake time led to a substantial decrease in the amplitude of the cortisol rhythm, while preserving the phase of the rhythm (Figure 7). The changes in the ACTH rhythm in response to CSR are much less pronounced (Figure S3) than those of cortisol.

In order to simulate the effects of evening light exposure on sleep timing and cortisol levels, the nighttime light intensity was varied from 0 to 40 lux. In general, exposure to evening light delayed both the peak of the circadian drive and sleep and wake timing which subsequently resulted in a delay in the cortisol peak without a discernable change in the amplitude of the rhythm. Using similar evening light exposure profiles to those use by Skeldon et al. [34] resulted in a delay in the cortisol rhythm of approximately 1–2 h depending on the evening light intensity. CSR in the presence of evening light resulted in a more pronounced dampening of the cortisol circadian rhythm (Figure S4). Our results suggest that this increased dampening of the cortisol rhythm might be related to the decrease in the amplitude of the intrinsic circadian drive when CSR is imposed together with low-intensity evening light exposure (Figure S5).

**Figure 8:**
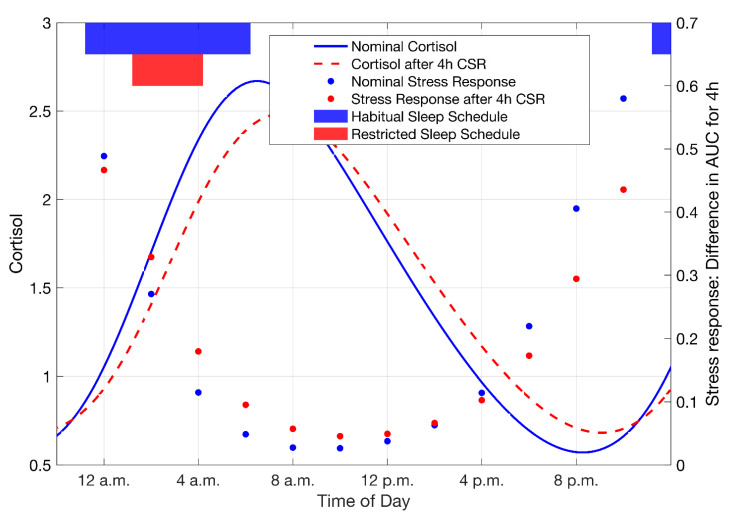
The influence of 4 h of CSR on the stress response of the HPA axis. The nominal cortisol profile (blue) in comparison to the cortisol rhythm after 4 h CSR (red). The timing of the sleep episodes is depicted by the blue (habitual) and red (CSR) bars at the top of the figure. The glucocorticoid acute stress response is calculated as the difference in AUC for 4 h between stressed and unstressed states for the two conditions. The dots depict the acute stress response, for the two conditions, at the indicated times at which the simulated acute stressor was administered.

Finally, we find that CSR alters the model-predicted acute stress responsivity of the HPA axis. In general, the model exhibits a time-of-day dependent response leading to a more robust response during the evening, which is in qualitative agreement with observations of the cortisol response in human subjects in response to administration of the bacterial endotoxin, lipopolysaccharide (LPS), a commonly studied prototypical immune stressor [39]. Model simulations predict that 4 h of CSR which results in a dampening of the cortisol circadian amplitude, results in a decrease in the time-of-day dependence (i.e., decreased peak response) of the stress responsivity relative to habitual sleep schedule conditions (Figure 8).

## 3. Discussion

Our simulations suggest that CSR alone (i.e., changes in sleep duration without any other potentially associated reasons for altering the HPA axis, such as stress or postural changes associated with the increased time awake) results in substantial alterations in the circadian dynamics of the HPA axis, with a dampening of the rhythms and alterations in the phase of the rhythm being the most prominent effects. While mild CSR (less than ~2 h per night) does not substantially alter glucocorticoid rhythmicity, model simulations suggest that CSR of greater than ~2 h per night (i.e., less than 6 h of sleep per night) might have physiologically relevant consequences. Experimental studies have explored the effects of 2–10 days of CSR on cortisol dynamics and related putative physiological consequences. One of the first systematic investigations of the effects of CSR on the HPA axis used a protocol involving six consecutive nights of 4 h in bed and then a sleep recovery period of 12 h in bed for six consecutive nights [14] in a cohort of eleven young men. The CSR (compared to end of recovery condition) had increased cortisol levels in the afternoon and early evening and the circadian cortisol profile had a shorter quiescent period (i.e., fraction of circadian period corresponding to low cortisol secretion) with a ~1.5 h delay in its onset. This was accompanied by a substantially slower rate of decrease in the saliva cortisol concentration between 4 p.m. and 9 p.m. A subsequent study by the same group investigating the influence of 8 h in bed found evening levels of cortisol to be intermediate between two conditions compared in the prior study i.e., 4 h in bed for six consecutive nights resulted in highest evening cortisol levels and 12 h in bed for six consecutive nights resulted in lowest evening cortisol levels [40]. Our model simulations are in qualitative agreement with these experimental observations: simulated CSR was associated with an increase in nadir levels, a delay in the phase of the cortisol rhythm and thus a lower rate of decline of the rhythm. Epidemiological studies have also found chronic “short sleepers” (less than 5 h of sleep, self-reported, and self-selected at home) to have higher nocturnal cortisol levels in comparison to chronic long sleepers as well as a lower rate of decrease of cortisol after the diurnal peak [41,42]. Findings from these early studies of HPA axis activity in conditions of sleep debt have been consistent with more recent studies. Leproult et al. [20,43] and Guyon et al. [21] studied the effects of one night of 4 h in bed and two consecutive nights of 4 h in bed, respectively, and found increased evening levels of cortisol as well as a ~1 h delay in the quiescent period. Thus, CSR appears to result in increased nadir levels of cortisol, which is qualitatively supported by our model simulations.

In addition to the increase in nadir levels, model simulations predict an accompanying decrease in the morning peak levels of cortisol. Four consecutive days of 3 h of sleep per night in 10 young men was associated with decreased morning levels of cortisol (measured at 7 a.m.) [44]. It is hypothesized that CSR might lead to an alteration in the negative feedback properties of the HPA axis. Our model simulations further support this hypothesis, as seen by the increase in the nadir levels of the glucocorticoid receptors. This increased negative feedback during the night might eventually lead to a decrease in morning levels of cortisol subsequently leading to the sustained dampening of the cortisol circadian rhythm.

The exact mechanisms by which CSR-induced changes in cortisol rhythmicity, i.e., the model predicted increase in evening cortisol levels and associated dampening of the rhythm, mediate pathophysiological metabolic changes have been difficult to determine. However, a number of laboratory-based studies have found that CSR is associated with moderate increases in afternoon and evening cortisol circadian rhythms (in qualitative agreement with model simulations) along with impaired glucose tolerance (quantified as the rate of decrease in glucose levels either after an IV or oral glucose tolerance test) and reduced insulin sensitivity [30,45,46]. For instance, Reynolds et al. [45] found that five nights of 4 h sleep resulted in higher cortisol afternoon cortisol levels and increase glucose as well as insulin levels. Buxton et al. [22] found that 1 week of 5 h in bed was also associated with impaired insulin sensitivity and increased evening cortisol levels, although changes in cortisol levels were not statistically significantly correlated to changes in insulin sensitivity at an individual level in this study. Recent evidence also suggests that dampened cortisol rhythms as well as elevated nocturnal cortisol levels are associated with increased incidence of obesity [47]. The characteristic features of increase in cortisol nadir [11,29] and phase delay [48] are reported to be associated with metabolic syndrome, indicating the functional linkage between CSR and metabolic disorders through circadian dysregulation of the HPA axis.

At a more mechanistic level, chronically dampened cortisol rhythms have been hypothesized to contribute to physiological dysregulation through imbalanced activation of MR and GR receptors [49]. MR and GR are found to mediate opposite effects on many target tissues, further underscoring the maintenance of an appropriate balance between MR- and GR-mediated effects [49]. We used our model to explore the potential changes in peripheral MR and GR activation as a result of CSR-induced changes in cortisol circadian rhythms. In general, the high-affinity MR is activated even at very low concentrations of cortisol. Therefore, at low cortisol levels, MR effects dominate as the majority of MR receptors are bound and transnucleated, while GR are still localized to the cytoplasm. On the other hand, GR translocates to the nucleus and exerts its genomic effects only above a threshold concentration, similar to that occurring during the diurnal cortisol peak or in response to stress exposure. The model-predicted nocturnal elevation of the circadian trough of the cortisol rhythm occurring in response to CSR causes a small but persistent increase in MR and GR activation during the cortisol trough, potentially resulting in a shift of the MR-GR balance with an excessive activation of MR-dependent mechanisms [49,50]. An imbalance in relative MR–GR activation, with a shift towards increased MR-dependent activation due to an elevated cortisol nadir is hypothesized to be a pathophysiological mechanism for metabolic disruption in obesity [50], insulin resistance [50], and diabetes [38]. Dampening of cortisol rhythms may also reduce its inhibitory effect on inflammatory pathway leading to increased levels of basal inflammatory response, indicating the association between increased inflammation and sleep restriction [51,52,53]. Further, dampened cortisol rhythms have been reported in post-traumatic stress disorder (PTSD) patients [54] along with sleep disturbances [55], increased inflammation [56], and metabolic disruption [57], suggesting inter-relationships among the HPA axis, sleep/wake cycle, inflammation, and metabolic disorder. Moreover, the model-predicted altered cortisol response to stress during nocturnal hours during 4 h CSR could further exacerbate the imbalance in relative receptor activation. Thus, the maintenance of appropriate homeostatic circadian rhythmicity in the HPA axis may not be only necessary for mounting an adequate response to stressors but also for the appropriate physiological regulation of glucocorticoid-sensitive target tissues across the body [13,14,58].

Using the modeling framework presented in this work, we further explored how CSR as a result of changes in wake time influence cortisol circadian rhythms in comparison to changes in sleep time. Interestingly, model simulations predict that the amplitude of the cortisol circadian rhythm is more sensitive to changes in wake time than to changes in sleep time. Put another way, “late-night” sleep loss results in a greater dampening of the cortisol rhythm in comparison to “early-night” sleep loss. Our results are in qualitative agreement with the study in which sleep loss during 3–6 a.m. was associated with a greater decrease in morning cortisol concentrations than sleep loss during 12 a.m.–3 a.m. [44].

Exposure to evening light results in a shift in both circadian rhythms and sleep/wake timing to later times of the day, and subsequently causes the circadian peak of the endogenous cortisol rhythm to be delayed by approximately 1 h (Figures S1 and S2) [34]. While we did not specifically explore the influence of social constraints on cortisol circadian rhythmicity, social interactions and/or other choices may impact sleep schedules and increase evening light exposure. Model simulations suggest that CSR can have amplified effects in the presence of evening light, with a more pronounced dampening of the cortisol circadian rhythm.

In conclusion, our model simulations suggest that CSR alone can cause substantial disruption of glucocorticoid circadian rhythmicity and HPA axis activity, which can potentially lead to detrimental metabolic and other physiological effects. The mathematical model presented in this work provides a mechanistically plausible framework for further exploration of the complex feedback dynamics between sleep and the HPA axis. The model presented here has a number of important limitations and potential areas for extension: (i) While our model only accounts for the unidirectional influence of sleep and intrinsic circadian drive on HPA axis rhythms, we envision future iterations of the model that account for the bidirectional influence of the HPA axis on sleep dynamics. (ii) We only consider relatively low levels of evening light exposure (up to 40 lux), which might not be representative of evening light intensities often experienced in modern environments and could be explored in future iterations. (iii) We consider simplistic photoperiods in our work, while 16 h light/8 h dark photoperiods that are more representative of those experienced by modern humans should be explored. (iv) Our model does not reproduce the observed cortisol awakening response [59]. The cortisol awakening response, which is a surge in cortisol production associated with the beginning of the wake episode/end of the sleep episode, occurs at a much faster timescale, on the order of that of ultradian frequency of cortisol pulsatility, which is not accounted for in our model. (v) The influence of sex- and age-related differences in HPA axis activity and the physiological sleep rhythms can be further accounted for in subsequent iterations of the model. (vi) The influences of other causes of perturbation and response of the HPA axis (e.g., stress and posture) should be added to the model. (vii) The mathematical model presented in this work can be expanded to explicitly account for the influence of cortisol on the dynamics of metabolic mediators such as glucose and insulin as has been shown by others [57,60,61,62,63]. Such extensions would enable further investigation of the interplay among timing of meals, sleep cycles, and light exposure, which are often perturbed together for extended periods in chronic shift workers. Thus, the inclusion of the interlinked influence of both circadian- and sleep-related stimuli on the dynamics of the HPA axis as well as other important metabolic mediators potentially enables a more complete investigative tool to study the inter-dependent regulation of sleep, stress and metabolism in health and disease.

## 4. Methods

### 4.1. Physiological Model of Sleep/Wake Regulation

The physiological model of the sleep/wake cycle combines two extensively validated mathematical models: (i) a model describing the switching between sleep and wake states in a manner dependent on circadian and homeostatic drives [64], respectively; (ii) a model describing entrainment dynamics of the circadian system [65], where the circadian oscillator is modeled as a van der Pol oscillator. This form of the mathematical model has been previously used to investigate the changes in sleep/wake regulation due to the influence of rotating and non-rotating shift-work schedules [66,67,68], internal desynchrony [69], exposure to evening artificial light and the influence of age-related physiological alterations in endogenous circadian rhythms [34]. Detailed descriptions of the model parameters are provided in Table S1.

Sleep and wake states are generated by the model as a result of mutual inhibition of between sleep-promoting (ventrolateral preoptic, VLPO) and wake-promoting (monoaminergic, MA) neurons [70]. The interactions between these neuronal populations are described by their mean electric potentials, V_v_ and V_m_, which represent the VLPO neuronal population and MA neuronal population, respectively (Equations (1) and (2)). The mean electric potential of the VLPO neurons is further induced by a homeostatic sleep drive, which is indicative of homeostatic sleep pressure, and increases monotonically during waking hours (Equation (3)). These mean electric potentials ultimate result in firing of the neuronal populations (Q_m,v_), with the mean firing rate of each population being described in Equation (5). The model describes a wake state when the firing rate of the wake-active MA population (Q_m_) is greater than the firing rate of the sleep-inducing VLPO population (Qv).

In addition to homeostatic sleep pressure effects, the VLPO population is regulated by the circadian clock [71] (Equation (5)). The circadian oscillator itself is modeled as a forced van der Pol oscillator, where the forcing represents a light intensity dependent signal (Equations (6)–(8)). The ability of light to entrain the circadian clock is further gated by sleep/wake state [34]. Further details regarding specific parameters in the model are described in the Supplementary Materials and Methods. Model parameters values for the physiological model of sleep/wake regulation were taken from previously published work by Skeldon et al. [34], based on the modified Philips Chen Robinson model. Nominal parameters for Equations (1)–(7), describing the dynamics of the VLPO, MA, and circadian and homoeostatic sleep pressure effects were chosen such that the wake state occurs approximately between 7 a.m. and 11 p.m., the circadian drive to sleep peaks around 4 p.m., and the homeostatic drive peaks towards the end of waking [72]. Parameter values for the light profiles (Equation (8)) were selected to generate light durations centered around noon.

For simplicity, the nominal simulated light profile used as a zeitgeber in the current study to entrain the endogenous circadian clock had an approximately 12 h photoperiod and was centered around 12 p.m. The intensity of the light schedule is qualitatively matched to light intensities used by Skeldon et al. [34], with a maximal intensity of approximately 1000 lux, and is similar to the light intensities humans are exposed to most frequently in modern environments composed of a mix of electrical lighting and natural light during the day [73]. In the nominal case, we assume an idealized situation where the light intensity approaches zero between approximately 6 p.m. and 6 a.m. While the current work for simplicity only considers an idealized symmetric 12 h light schedule centered around noon, similar to that used previously to model sleep schedules prevalent in hunter gatherer societies [34], the model has the flexibility to represent light schedules more typically observed by humans in modern day societies. In the nominal case, the light profile was simulated using Equation (7) such that it approximately alternated between complete brightness and complete darkness. In order to simulate the effects of evening light exposure on cortisol levels, the nighttime light intensity was varied within 0–40 lux. In such conditions, the time when lights are completely OFF (0 lux) is determined by when the model switches between wake and sleep states.

### 4.2. Semi-Mechanistic Model of the HPA Axis

The primary mediators of the HPA axis are represented in this work by nonlinear ordinary differential equations (ODEs) that form a limit-cycle type Goodwin oscillator [74]. The Goodwin oscillator represents a phenomenological prototypical biological oscillator, initially devised to show the possibility of self-sustained oscillations in a genetic circuit with a simple delayed negative feedback loop architecture. The basic structure of the Goodwin oscillator adapted to model the circadian oscillations in the HPA axis was initially devised by Sriram et al. [75] and was modified subsequently to account for the entrainment of the HPA axis rhythms by light via the SCN in both nocturnal and diurnal species [76,77].

Briefly, according to the scheme of equations presented here, CRH induces the release of ACTH, which subsequently induces the release of cortisol (Equations (9)–(11)). The synthesis of CRH is described by zero-order kinetics (k_p1_), while the synthesis of ACTH and cortisol is described by first-order kinetics (k_p2_ and k_p3_, respectively). The degradation of each of the three mediators is described by Michaelis–Menten kinetics. The use of Michaelian kinetics in our model scheme precludes the use of unreasonably high values of the Hill coefficient [77,78], generally interpreted as the number of ligand subunits cooperatively binding to a receptor. Moreover, CRH, ACTH, and the glucocorticoids all undergo enzymatic metabolism and degradation [79,80,81]. We further account for glucocorticoid receptor (GR)-mediated pharmacodynamics, as adapted from Ramakrishnan et al. [82] (Equations (12)–(15)). The binding of released cortisol (CORT) to cytosolic GR, modeled using second-order kinetics (Equation (14)), results in the formation of the receptor–glucocorticoid complex (DR). DR(N) represents the nuclear activated receptor–glucocorticoid complex assumed to be responsible for the receptor-mediated effects of glucocorticoids. The receptor mediated inhibition of CRH and ACTH by the glucocorticoids is described in Equations (9) and (10), respectively, and accounts for the negative feedback loop of the HPA axis. K_p1_ and K_p2_ are the inhibition constants and are indicative of the strength of inhibition in the negative feedback loop, with smaller values indicative of stronger negative feedback. Furthermore, Equation (12) describes the negative regulation of DR(N) on the transcription of GR mRNA.

Finally, the model accounts for cortisol binding and activating its two receptors in the periphery, the mineralocorticoid (MR) and the glucocorticoid (GR) receptors, adapted from previously published models [60]. Receptor activation results in hyperphosphorylation, and receptor conformational changes [82]. We assume hyperphosphorylation to be the rate-determining step [83] for both receptors (Equations (16) and (19)). Assuming that the phosphorylation and degradation steps for both receptors exhibit saturation, we described these processes with Michaelis–Menten kinetics. Production of MR and GR is regulated by their base production rates (k_MR_ and kGR), Michaelis constants for production (K_F,MR_ and K_F,GR_) and the concentration of inactivated receptors, computed as the difference between the total receptor concentration (MR_T_ and GR_T_) and the phosphorylated/activated receptor (MR and GR). Additionally, a cortisol mediated indirect mechanism of activation is modeled with efficiency constants of stimulation represented by the k_F, MR_ and k_F, GR_ parameters. Michaelis constants of cortisol induced activation are described by K_F,MR_ and K_F,GR_. The translocated nuclear receptor–glucocorticoid complexes (Equations (17),(18),(20) and (21)) are assumed to their effects via binding to their respective glucocorticoid responsive elements (GREs). The difference in the binding affinities of cortisol to these receptors [84] was modeled by setting the Michaelis constant of the mineralocorticoid receptor (K_MR_) to a lower value than that of glucocorticoid receptor (K_GR_). Model parameters for the HPA axis (Equations (9)–(11)) were selected such that HPA axis rhythm in the absence of the entraining effects of light adopts a period of slightly greater than 24 h (24.1–24.5 h) [85], while, in the presence of the nominal light schedule, cortisol adopts a robust rhythm with a peak around the beginning of the wake state in qualitative agreement with experimental observations (Figure S3). Equations (12)–(21) were parameterized based on a corticosteroid pharmacodynamic model calibrated to experimental data on corticosterone and corticosteroid receptor dynamics after methylprednisolone administration [82,86].

The physiological model of the sleep/wake states is coupled to the model of the HPA axis by accounting for the influence of both the circadian drive (representative of the influence of the central circadian pacemaker in the SCN) and sleep/wake drive on the synthesis of CRH [10]. The central circadian rhythm is assumed to drive the oscillations of the HPA axis mediators by regulating the synthesis of CRH (Equation (9)). The effect of the sleep/wake cycle is described as an inhibitory influence assumed to regulate the degradation of CRH. Several studies suggest that slow-wave sleep (one of the scored sleep stages) has a mild inhibitory influence on the HPA axis. For instance, CRH pulsatility is inversely associated with cycles of slow-wave sleep. Furthermore, studies have found that cortisol levels are lower after daytime naps [10,13,20]. Thus, we hypothesize that the sleep inhibits the HPA axis by increasing the clearance of CRH (Equation (9)). It should be noted that cortisol exhibits prominent pulsatile ultradian rhythms, which are not captured by the dynamics of our model. Nevertheless, previously published models of the HPA axis based on a similar framework that have primarily studied the circadian dynamics of this system have yielded insightful physiologically findings in understanding the mechanisms contributing to disruption in cortisol circadian rhythms in PTSD and depression [60,63,75].

### 4.3. Sleep/Wake Schedules

Several sleep schedules were used in the study. (1) In the nominal case, in the absence of CSR, sleep and wake times are determined by the entirely by the dynamics of the model given the nominal parameterization (Table S1), resulting in an approximately 8 h sleep schedule with a sleep time around 11 p.m. and a wake time of around 7 a.m. (2) To simulate the effects of CSR, three different sleep schedules were used to explore the differential effects of altering the wake time and sleep time on the circadian rhythm of cortisol: (2.1) delaying the sleep time and advancing by an equal duration the wake time relative to the habitual sleep and wake time determined by the dynamics of the model, which results in a sleep schedule that is centered around the same time of day as the habitual sleep Schedule I; (2.2) delaying the sleep time while maintaining the habitual wake time; (2.3) advancing wake time while maintaining habitual sleep time.

### 4.4. Influence of CSR on Acute Stress Response

Given that a primary function of the HPA axis is to appropriately respond to acute stressors and mount an adequate stress response, we explored whether CSR alters the stress responsivity of the HPA axis. The simulated acute stressor results in a transient increase in the rate of CRH synthesis. This transient increase in CRH synthesis is a simplified general representation that might simulate the effect of various acute stressors, both psychological, such as restraint stress, and physiological, such as bacterial lipopolysaccharide (LPS) exposure. The cortisol response to the acute stressor is characterized by the difference in area under the curve (AUC) between the respective cortisol profiles with and without exposure to the acute stressor for 4 h from the time of in silico exposure to the acute stressor.

Circadian and Sleep/Wake Dynamics

(1) $$\frac{dV_{v}}{dt}=\left(-V_{v}-v_{m}Q_{m}+D_{v}\right)/\tau_{v}$$

(2) $$\frac{dV_{m}}{dt}=\left(-V_{m}-v_{m_{v}}Q_{v}+D_{m}\right)/\tau_{m})$$

(3) $$\chi \frac{dH}{dt}=-H+\mu Q_{m}$$

(4) $$D_{v}=A_{v}+v_{vc}C\left(t\right)+v_{vh}H\left(t\right)\;$$

(5) $$Q_{v,m}=\frac{Q_{max}}{1+exp\left(-V_{v,m}-\theta \right)/\sigma )}\;$$

(6) $$\kappa \frac{dx}{dt}=\gamma \left(x-\frac{4x^{3}}{3}\right)-y(\left(frac24f\tau_{c}{)}^{2}+\kappa B\right)\;\kappa \frac{dx}{dt}=x+B\;\frac{dn}{dt}=\lambda (\alpha_{0}{\left(\frac{I}{I_{0}}\right)}^{p}\left(1-n\right)-\beta n)$$

(7) $$B=\alpha_{0}{\left(\frac{I}{I_{0}}\right)}^{p}\left(1-n\right)\left(1-bx\right)\left(1-by\right)\;I=H(Q_{m}-Q_{th})I(t)\;I(t)=l_{2}+\frac{\left(l_{1}-l_{2}\right)}{2}\tanh(c(t-s_{1}))-\tanh(c(t-s_{2}))$$

(8) C(t)=0.55(1+0.8y−0.47x)

HPA Axis Dynamics

(9) $$\frac{dCRH}{dt}=\frac{k_{p1}\cdot K_{p1}\cdot \left(1+\frac{SCN_{drive}}{1+SCN_{drive}}\right)}{K_{p1}+GR_{bound}\left(N\right)}-V_{d1}\cdot \frac{CRH\cdot \left(1+k_{s}\cdot sleep\right)}{K_{d1}+CRH}$$

(10) $$\frac{dACTH}{dt}=\frac{k_{p2}\cdot K_{p2}CRH}{K_{p2}+DR\left(N\right)}-V_{d2}\cdot \frac{ACTH}{K_{d2}+ACTH}$$

(11) $$\frac{dCORT}{dt}=k_{p3}\cdot ACTH-V_{d3}\cdot \frac{CORT}{K_{d3}+CORT}$$

(12) $$\frac{dGR_{mRNA,cen}}{dt}=k_{syn_{GRm,cen}}\cdot \left(1-\frac{DR{\left(N\right)}_{cen}}{IC_{50_{GRm,cen}}+DR{\left(N\right)}_{cen}}\right)-k_{deg}\cdot GR_{mRNA,\;cen}$$

(13) $$\frac{dGR_{cen}}{dt}=k_{syn,GR,cen}\cdot GR_{mRNA,cen}+r_{f}\cdot k_{re}\cdot DR\left(N\right)-k_{on}\cdot \left(CORT\right)\cdot GR_{cen}-k_{deg,GR,cen}\cdot GR_{cen}$$

(14) $$\frac{dDR_{cen}}{dt}=k_{on}\cdot \left(CORT\right)\cdot GR_{cen}-k_{T}\cdot DR_{cen}$$

Peripheral Glucocorticoid Receptor Dynamics

(15) $$\frac{dMR}{dt}=k_{MR}\left(\;\frac{\left(1+\frac{k_{F,MR}\cdot CORT}{K_{F,MR}+CORT}\right)\left(MR_{T}-MR\right)}{K_{MR}+MR_{T}-MR}\right)-\frac{k_{MR,deg}\cdot MR}{K_{MR,deg}+MR}-k_{b,MR}\cdot CORT\cdot MR+k_{r,MR}\cdot FMR\left(N\right)$$

(16) $$\frac{dFMR}{dt}=k_{on,MR}CORT\cdot MR-k_{T,MR}FMR$$

(17) $$\frac{dFMR\left(N\right)}{dt}=k_{T,MR}FMR-k_{re,MR}FMR\left(N\right)$$

(18) $$\frac{dGR}{dt}=k_{GR}\left(\frac{\left(1+\frac{k_{F,GR}\cdot CORT}{K_{F,GR}+CORT}\right)\left(GR_{T}-GR\right)}{K_{GR}+GR_{T}-GR}\right)-\frac{k_{GR,deg}\cdot GR}{K_{GR,deg}+GR}-k_{b,GR}\cdot CORT\cdot GR+k_{r,GR}\cdot FGR\left(N\right)$$

(19) $$\frac{dFGR}{dt}=k_{on,GR}CORT\cdot GR-k_{T,GR}FGR$$

(20) $$\frac{dFGR\left(N\right)}{dt}=k_{T,GR}FGR-k_{re,GR}FGR\left(N\right)$$

## Data Availability

All data are available within the article.

## Supplementary Materials

The following are available online at https://www.mdpi.com/article/10.3390/metabo11080483/s1, Figure S1: (A): Light schedule with evening light exposure (40 lux) in comparison to the nominal light schedule with no evening light [maximal light intensity of 1000 lux after simulated sunrise (6AM) and a minimal light intensity of 20 lux after the simulated sunset (6PM)] (B) the neuronal firing rates for the light schedule with evening light exposure (40 lux and for the nominal schedule (no evening light), respectively. The (C) circadian drive and cortisol circadian rhythm for the light schedule with evening light exposure (40 lux and for the nominal schedule (no evening light), respectively, Figure S2: Cortisol circadian rhythms during CSR upon exposure to evening light [maximal light intensity of 1000 lux during after simulated sunrise (6AM) and a minimal light intensity of 40 lux after the simulated sunset (6PM)] as a result of the sleep schedules shown in Figure S3: (A) changing both wake and sleep onset times (a) (B) changing only onset sleep time (b1) (C) changing only wake onset time t. (b2) Left panels: Simulated CSR schedules used. Middlepanels: Cortisol circadian rhythms with increasing levels of CSR. Color of line reflects schedule in left panels. (C) Phase of the cortisol rhythm with different levels of CSR. Color of line reflects schedule in left panels, Figure S3: (A) Simulated 48 hours of CRH (blue) and ACTH (red) profiles under the nominal light schedule [maximal light intensity of 1000 lux after simulated sunrise (6AM) and a minimal light intensity of 0 lux after simulated sunset (6PM)] and habitual sleep schedule. Timing of sleep (black shaded bars at top of panels) and light-dark (white and grey shaded regions respectively) are indicated as in Figure 2. (B) Nominal cortisol rhythms qualitatively matched to mean normalized experimental cortisol observations from [1], Figure S4: ACTH circadian rhythms in response to varying durations of chronic sleep restriction as a result of (A) changing both wake and sleep onset times (condition a) (B) changing only sleep onset time (condition b1) (C) changing only wake onset time (condition b2), Table S1: List of parameters in the model.
