# Supplementary material for: Modeling the Influence of Chronic Sleep Restriction on Cortisol Circadian Rhythms, with Implications for Metabolic Disorders
Source: Metabolites. 2021 Jul 27;11(8):483. doi: 10.3390/metabo11080483 (PMC8400645; doi:10.3390/metabo11080483)
Supplement: Supplementary file 1 [file metabolites-11-00483-s001.zip › Supplementary_Materials/RRao_20210525_Supplementary_Materials_Metabolites_V5.docx]

# Supplementary Materials and Methods

## Supplementary Figures

Figure S1: A: Light schedule with evening light exposure (40 lux) in comparison to the nominal light schedule with no evening light [maximal light intensity of 1000 lux after simulated sunrise (6AM) and a minimal light intensity of 20 lux after the simulated sunset (6PM)] B) the neuronal firing rates for the light schedule with evening light exposure (40 lux and for the nominal schedule (no evening light), respectively. The C) circadian drive and cortisol circadian rhythm for the light schedule with evening light exposure (40 lux and for the nominal schedule (no evening light), respectively.

Figure S2: Cortisol circadian rhythms during CSR upon exposure to evening light [maximal light intensity of 1000 lux during after simulated sunrise (6AM) and a minimal light intensity of 40 lux after the simulated sunset (6PM)] as a result of the sleep schedules shown in Figure S3 – A) changing both wake and sleep onset times (a) B) changing only onset sleep time (b1) C) changing only wake onset time t.(b2) Left panels: Simulated CSR schedules used. Middle panels: Cortisol circadian rhythms with increasing levels of CSR. Color of line reflects schedule in left panels. C) Phase of the cortisol rhythm with different levels of CSR. Color of line reflects schedule in left panels


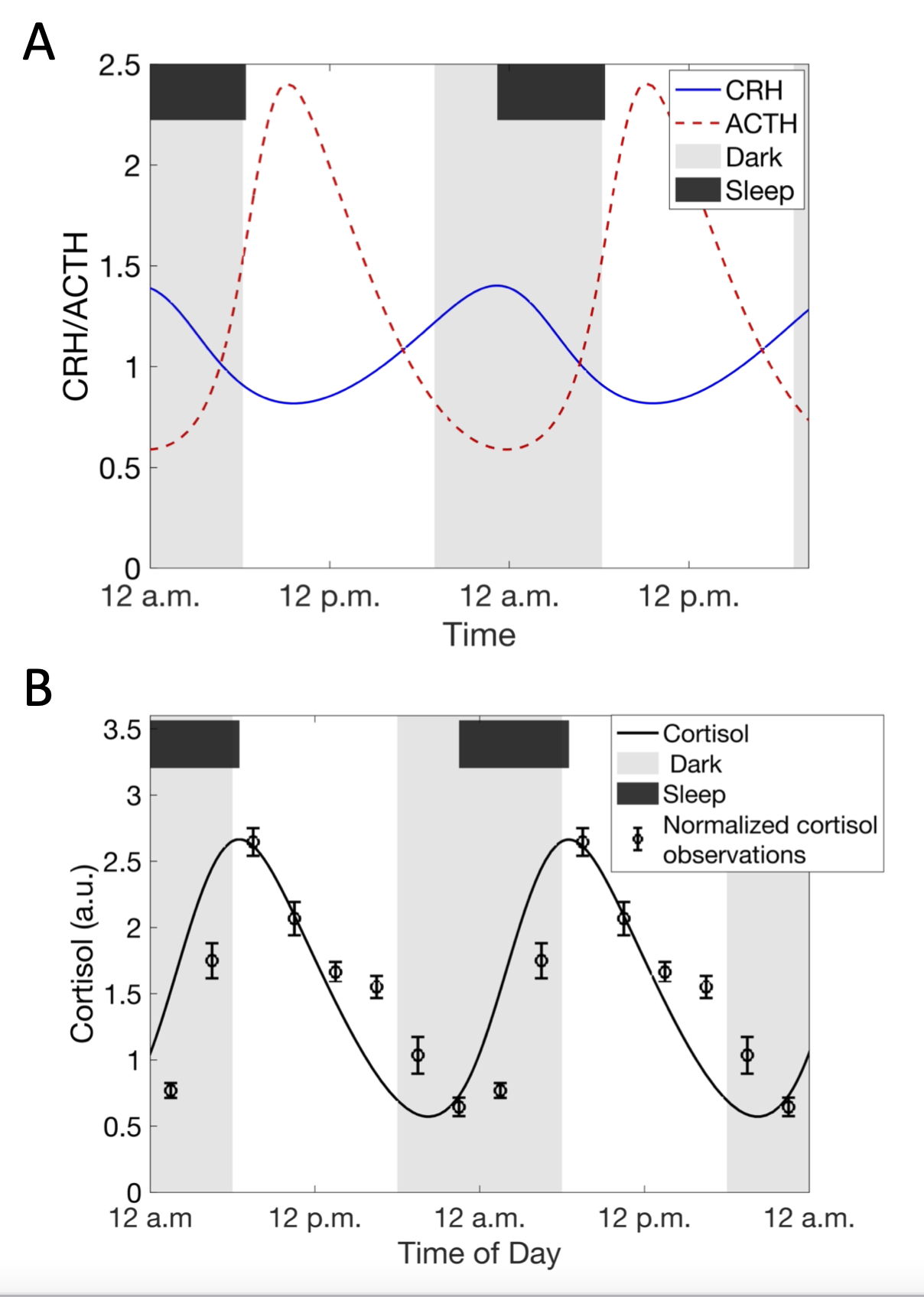


Figure S3:A) Simulated 48 hours of CRH (blue) and ACTH (red) profiles under the nominal light schedule [maximal light intensity of 1000 lux after simulated sunrise (6AM) and a minimal light intensity of 0 lux after simulated sunset (6PM)] and habitual sleep schedule. Timing of sleep (black shaded bars at top of panels) and light-dark (white and grey shaded regions respectively) are indicated as in Figure 2. B) Nominal cortisol rhythms qualitatively matched to mean normalized experimental cortisol observations from [1]

Figure S4: ACTH circadian rhythms in response to varying durations of chronic sleep restriction as a result of A) changing both wake and sleep onset times (condition a) B) changing only sleep onset time (condition b1) C) changing only wake onset time (condition b2).

## Supplementary Tables

Table S1: List of Parameters in the Model.

| **HPA Axis Parameters** | | |
| --- | --- | --- |
| Parameter | Value | Description |
| $k_{p1}$ | 0.7567 µMh^-1^ | Estimated, zero order synthesis rate constant of CRH |
| $V_{d1}$ | 0.4576 µMh^-1^ | Estimated, first order rate constant for CRH degradation |
| $K_{d1}$ | 1.9627 µM | Estimated, Michaelis-Menten constant for CRH degradation |
| $k_{p2}$ | 0.6171 µMh^-1^ | Estimated, first order rate constant for synthesis of ACTH |
| $V_{d2}$ | 0.5129 µMh^-1^ | Estimated, first order rate constant for degradation of ACTH |
| $K_{d2}$ | 0.3096 µM | Estimated, Michaelis-Menten constant for ACTH degradation |
| $k_{p3}$ | 0.7665 µM | Estimated, first order rate constant for synthesis of CORT |
| $V_{d3}$ | 0.3618 µMh^-1^ | Estimated, first order rate constant for CORT degradation |
| $K_{d3}$ | 0.4695 µM | Estimated, Michaelis-Menten constant for CORT degradation |
| $GR(0)$ | 540.7 nmol L^−1^ mg protein^−1^ | Initial GR content, [2] |
| ${GR}_{mRNA}(0)$ | 25.8 fmolg^−1^ | Initial GR mRNA content, [2] |
| $k_{syn_{GRm}}$ | 2.9 fmolg^−1^ h^−1^ | Zero order rate constant for synthesis of GR mRNA, [2] |
| $r_{f}$ | 0.49 | GR recycle fraction from nucleus to cytoplasm, [2] |
| $k_{re}$ | 0.57 h^-1^ | Rate of GR recycling from nucleus to cytoplasm, [2] |
| $k_{on}$ | 0.00329 L nmol^−1^ h^−1^ | Second-order rate constant for CORT-GR binding, [2] |
| $k_{deg,GRm}$ | $k_{syn_{GRm}}/{GR}_{mRNA}(0)$ | First-order rate constant for degradation of GR mRNA, [2] |
| $k_{deg,GR}$ | 0.0572 h^-1^ | First order rate constant for degradation of GR, [2] |
| $k_{syn,GR}$ | $GR\left( 0 \right).k_{deg,GR}/ {GR}_{mRNA}(0)$ | First order rate constant for synthesis of GR, [2] |
| $k_{T}$ | 0.63 h^-1^ | Rate of GR translocation from cytoplasm to nucleus, [2] |
| $k_{imp}$ | 0.5 | Strength of ACTH impulse |
| $k_{stress.out}$ | 6.79 h^-1^ | Rate constant for clearance of stressor |
| $k_{s}$ | 40 | Strength of induction of CRH production by stressor |
| **Physiological Sleep Model Parameters** | | |
| $Q_{max}$ | 100s^-1^ | Maximum possible neuronal firing rate |
| θ | 10 | Value of the potential when half the maximal firing rate is attained |
| ν_mv_ | 1.8mVs | Influence of VLPO voltage on firing of MA population |
| ν_vm_ | 2.1mVs | Influence of MA voltage on firing of VLPO population |
| τ_m_ | 10s | Time-scales of neuronal parameters |
| µ | 3.8nMs | Rate constant for production of somnogenic mediator |
| A_m_ | 1.3mV | Drive to wake promoting neurons |
| τ_v_ | 10s | Time-scales of neuronal parameters |
| Q_th_ | 1s^-1^ | Threshold for light-induced firing of photoreceptive neurons |
| σ’ | 3mV | Width of sigmoid describing the neuronal firing rates |
| ν_vc_ | 2.5mV | Influence of circadian drive on sleep promoting neurons |
| Av | -10.2mV | Constant background inhibitory input to sleep-promoting neurons |
| χ | 45h | Inverse of rate constant for homeostatic process |
| κ | 12/π h | Inverse of rate constant for circadian process |
| τc | 24.1h | Intrinsic circadian period |
| I0 | 9500 lux | Saturation light intensity |
| p | 0.6 | Exponent of influence of photoreceptor saturation on circadian rhythm |
| γ | 0.23 | Coefficient for van der Pol oscillator state equation (x) |
| k | 0.55 | Constant determining forcing of light on circadian oscillator |
| β | 0.013 | Photoreceptor decay parameter |
| f | 0.99669 | Correction factor for entrainment period of van der Pol oscillator |
| α0 | 0.16 | Constant for light forcing function |
| b | 0.4 | Constant determining phase sensitivity of light saturation |

References

1. Roelfsema, F.; van Heemst, D.; Iranmanesh, A.; Takahashi, P.; Yang, R.; Veldhuis, J.D. Impact of age, sex and body mass index on cortisol secretion in 143 healthy adults. *Endocr Connect* **2017**, *6*, 500-509, doi:10.1530/EC-17-0160.

2. Ramakrishnan, R.; DuBois, D.C.; Almon, R.R.; Pyszczynski, N.A.; Jusko, W.J. Fifth-generation model for corticosteroid pharmacodynamics: application to steady-state receptor down-regulation and enzyme induction patterns during seven-day continuous infusion of methylprednisolone in rats. *J Pharmacokinet Pharmacodyn* **2002**, *29*, 1-24.
